# Tardigrade Dsup extends *C. elegans* life span by impeding mitochondrial respiration and promoting oxidative stress resistance

**DOI:** 10.1126/sciadv.adx9669

**Published:** 2025-10-31

**Authors:** Myriam Richaud, Rohit Shrivastava, Dimitris Xirodimas, Simon Galas, Aymeric Bailly

**Affiliations:** ^1^ IBMM, University of Montpellier, CNRS, ENSCM, Montpellier, France.; ^2^ CRBM, University of Montpellier, CNRS UMR5237, BIOLuM, Montpellier, France.

## Abstract

Tardigrades survive extreme environments partly through the damage suppressor (Dsup) protein, which protects DNA from ionizing radiation and oxidative stress. Dsup is largely unstructured but binds nucleosomes to shield DNA from damage. To investigate its protective role in a whole organism, we expressed the *Ramazzottius varieornatus* Dsup gene in the nematode *Caenorhabditis elegans*. Transgenic worms tolerated x-ray exposure and oxidative stress without apparent toxicity and exhibited a notable extension of life span. This effect was independent of the canonical DAF-2/DAF-16 longevity pathway and mitochondrial dynamics. Instead, Dsup expression markedly reduced mitochondrial respiration, providing a plausible mechanism for enhanced oxidative stress resistance and extended longevity. Our findings demonstrate that Dsup can confer stress resistance and longevity benefits across species, highlighting a unique protective strategy with potential implications for understanding aging and developing stress-resilient organisms.

## Teaser

Tardigrade Dsup extends *C. elegans* life span by reducing mitochondrial activity and enhancing stress resistance.

## INTRODUCTION

Tardigrades are small invertebrates (0.1 to 1.2 mm) that inhabits oceans, mountains, freshwater, mosses, and lichens (*1*). A surrounding film of water is needed to allow the activity of terrestrial tardigrades. However, when the film of water evaporates, tardigrades lose body water and enter a state of resistance called anhydrobiosis (*1*, *2*). The anydrobiotic state allows tardigrades to cope with extreme treatments like high temperature (*3*), high pressure (*4*), and radiations (*5*, *6*), as well as after a direct exposure to space radiation (*7*). The dehydration processes, per se, can induce DNA damage, and it has been observed that organisms like chironomids, which are also able to enter anhydrobiosis, showed nearly 40 to 50% of fragmented nuclear DNA in the course of the anhydrobiosis process (*8*), while one of the tardigrade species most resistant to the desiccation (*Milnesium inceptum*) showed 2% of fragmented DNA only (*9*). These observations led to the proposal that tardigrades may have a molecular equipment able to protect DNA from molecular damages induced by the dehydration (*5*). The first genome sequence from the tardigrade species *Hypsibius exemplaris* and *Ramazzottius varieornatus* (*R. varieornatus*) has unveiled tardigrade-unique genes like the Damage suppressor Dsup (Rv Dsup) (*10*–*12*). The heterologous expression of the Rv Dsup gene in human cells and plants has been shown to increase protection against x-ray radiation (*13*, *14*) as well as to promote cell survival upon oxidative stress induced by hydrogen peroxide (H_2_O_2_) treatment. A first indication about the interaction of the Rv Dsup protein with DNA (*13*) was then extended by a report showing its preferential association with in vitro assembled nucleosomes and the preservation of DNA fragmentation induced by treatment with hydroxyl radical oxygen (*15*). The study of the activity of the tardigrade Dsup gene has focused primarily on its intrinsic properties of protecting nucleic acids, cultured cells, or plant against acute oxidative stress or x-ray radiation (*13*–*15*).

The study investigates whether the tardigrade *R. varieornatus* damage suppressor protein (Dsup) can confer stress resistance and extend life span in *Caenorhabditis elegans*. By expressing Dsup in the nematode, the work aims to determine its protective effects against x-ray and oxidative stress while assessing possible toxicity. It further seeks to disentangle the molecular pathways underlying life span extension, testing the role of canonical regulators such as DAF-2/DAF-16 and mitochondrial dynamics. Last, the article explores reduced mitochondrial respiration as a potential mechanism linking Dsup to oxidative stress resistance and longevity.

## RESULTS

### Dsup expression in *C. elegans*

**Fig. 1.**
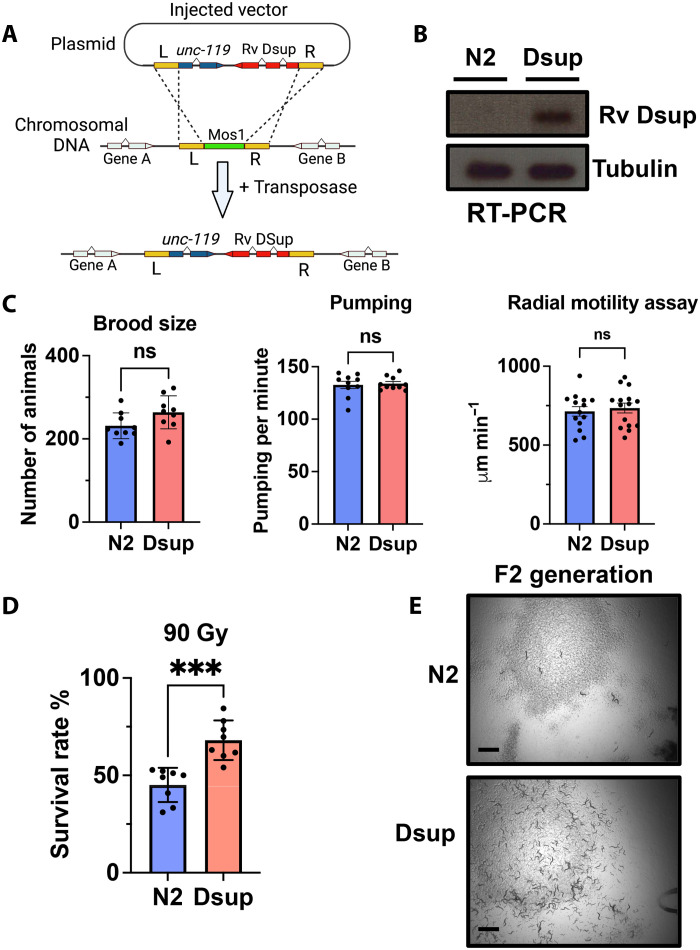
Heterologous expression of Rv Dsup in *C. elegans* does not cause overall toxicity and confers protection from ionizing radiation. (**A**) Single-copy insertion of Rv Dsup in the *C. elegans* genome by MosSCI. The MosSCI approach uses the Mos1 transposase to produce double-strand break, which is repaired using homologous sequences (L and R) from the injected vector. The integrated lines are selected by the UNCoordinated phenotype rescue following *unc-119* expression. (**B**) Specific Rv Dsup gene expression validation by RT-PCR. Total RNA was extracted from unsynchronized worm populations, followed by cDNA synthesis. Gene-specific primers for Rv Dsup were used to confirm expression, while *tbg-1* (encoding tubulin) served as an internal control. (**C**) Expression of Rv Dsup does not affect key physiological traits in *C. elegans*. No significant (ns) differences were observed in brood size (*n* = 10), pharyngeal pumping rate (*n* = 9), or motility (*n* = 17) between Rv Dsup–expressing and control worms. Data are presented as means ± SEM. Statistical signifance was determined using a two-tailed Student’s *t* test. (**D**) Heterologous expression of Rv Dsup enhances survival following ionizing radiation exposure. Young adult worms (24-hour post-L4 larvae stage, *n* = 8) were irradiated, and survival rate in the following generation was assessed (left). Data are presented as means ± SEM with statistical significance determined by a two-tailed Student’s *t* test. (**E**) Representative images of worm populations 5 days postirradiation (right) show that wild-type (N2) worms exhibited severe growth impairment, whereas Rv Dsup–expressing worms displayed improved survival. Scale bar, 2 mm.

To investigate the role of Dsup in a physiological context, we used the Mos single-copy insertion (MosSCI) method (*16*) to generate a stable single-copy insertion of the Rv Dsup gene, driven by the germline-specific spn-4 promoter in *C. elegans* (Fig. 1A). We assessed the correct integration by sequencing and the Rv Dsup gene expression by reverse transcription polymerase chain reaction (RT-PCR) (Fig. 1B) and selected one line (COP2427) that we will refer to as Dsup. Overall, we did not observe any negative effect of Dsup expression on *C. elegans* fitness as both wild-type N2 and Dsup lines display similar brood size, pharyngeal pumping, and motility under homeostatic conditions (Fig. 1C).

### Dsup enhances resistance to x-ray–induced DNA damage

To evaluate the impact of Dsup expression under stress conditions, we exposed wild-type N2 and Dsup-expressing worms to 90 gray of x-ray radiation and assessed the embryonic lethality in the following generation as a measure of genotoxic sensitivity. The presence of the Dsup gene significantly enhances survival, consistent with previous findings in cultured mammalian cells demonstrating Dsup’s protective effect against x-ray exposure (Fig. 1D) (*13*). This also confirms the functionality of our genetic construct. The protective effect observed in the F1 generation is even more pronounced in the F2 generation, where N2 worms exhibit severely impaired propagation compared to Dsup-expressing worms (Fig. 1E). These results establish that the tardigrade Dsup gene can be successfully expressed in a multicellular organism and provides protection against x-ray–induced DNA damage at the whole-organism level.

### Dsup provides strong protection against oxidative stress

**Fig. 2.**
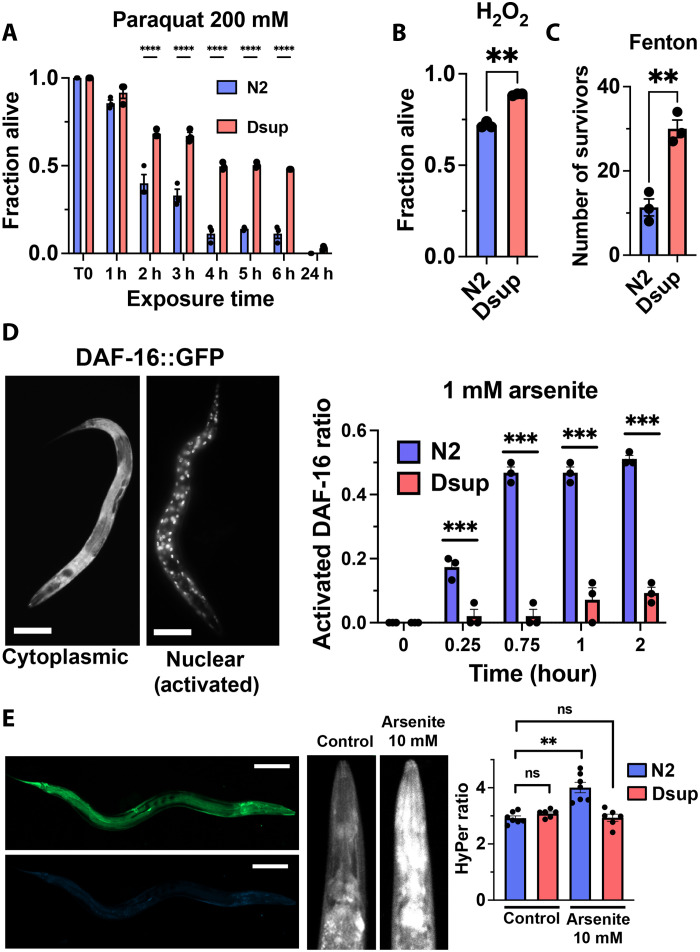
Dsup expression provides protection against oxidative stress in *C. elegans*. (**A**) Fraction of surviving animals of the indicated genotype following treatment of L4 larvae stage worms (*n* = 50 in triplicate) with 200 mM of paraquat (methyl viologen). h, hours. Data are means ± SEM. *P* values were determined using two-tailed Student’s *t* test. (**B**) Fraction of surviving worms of the indicated genotype (*n* = 100, in triplicate) treated with 1 mM of H_2_O_2_ for 30 min at 20°C. Data are means ± SEM, *P* values were determined using two-tailed Student’s *t* test. (**C**) Number of surviving animals after exposure to the Fenton’s reaction adapted for *C. elegans* (*58*). Data are means ± SEM, *n* = 50, *P* values were determined using two-tailed Student’s *t* test. (**D**) Dsup expression delays DAF-16 activation induced by arsenite exposure. DAF-16 activation ratio was assessed by measuring the fraction of animals with DAF-16::GFP nuclear versus cytoplasmic localization exposed to 1 mM of arsenite. Representative pictures of cytoplasmic and nuclear localization are shown on the left (bar, 100 μm). Data are means ± SEM. *P* values were determined using two-tailed Student’s *t* test, *n* = 30, in triplicate. (**E**) The HyPer biosensor indicates a reduced overoxidized ratio (HyPer Ratio) in Dsup upon treatment with arsenite. Representative picture of a whole worm is shown on the left for 488- and 405-nm wavelength (green and blue channel respectively); bar, 100 μm. The center panel shows a representative worm pharynx at 488 nm after arsenite treatment. The right panel depicts the graphical representation of the 405 over 488 nm ratio in N2 wild-type and Dsup worms upon arsenite treatment (*n* = 7). Data are means ± SEM, *P* values were determined using two-tailed Student’s *t* test. *P < 0.1, **P < 0.05, ***P < 0.001.

Because Dsup expression has been associated with resistance toward oxidative stress in human cultured cells (*13*), we asked whether Dsup expression shows a similar protective effect at the whole-organism level in *C. elegans*. We exposed wild-type N2 and Dsup-expressing worms to paraquat (methyl viologen), an oxidative stress–inducing compound, and monitored their survival over time. While only a slight difference was observed after 1 hour, a notable effect emerged at 4 hours: Nearly all N2 control worms had died, whereas 50% of Dsup-expressing worms remained alive, with this difference persisting up to 6 hours (Fig. 2A). We validated Dsup specificity by depleting its expression through RNA interference (RNAi) (fig. S1A). This result demonstrates that Dsup provides a robust protection against oxidative stress in a multicellular organism, extending beyond its previously reported effects in cultured cells. To further confirm this resistance, we examined the response to another oxidative stressor, H_2_O_2_. Endogenously produced as a byproduct of mitochondrial respiration via the reduced form of nicotinamide adenine dinucleotide phosphate oxidase (*17*), H_2_O_2_ generates highly reactive hydroxyl radicals (·OH) through Fenton’s reaction with cellular Fe^2+^ ions. Wild-type and Dsup-expressing worms were subjected to an acute 30-min treatment with 1 mM H_2_O_2_, after which survival was assessed. Dsup expression significantly increased survival under these conditions (Fig. 2B), further confirming its role in oxidative stress resistance. To directly recapitulate reactive radical generation in cells, we adapted Fenton’s reaction for *C. elegans*. Briefly, worms were incubated in a test tube with a ferric solution droplet under the tube cap. The reaction was initiated by a brief spin and terminated by adding a large buffer volume. Similar to paraquat and H_2_O_2_ exposure, the hydroxyl radicals (·OH) generated by Fenton’s reaction were highly toxic, leading to severe lethality in N2 worms (Fig. 2C). Dsup expression significantly increased survival, indicating enhanced resistance to hydroxyl radical–induced oxidative damage. Collectively, these findings demonstrate that Dsup protects against oxidative stress from multiple sources in a whole multicellular organism, reinforcing its role beyond human cell culture systems.

### Dsup protects by reducing ROS

To account for the Dsup protection against oxidative stress, we considered two possible mechanisms: (i) Dsup enhances cellular resistance to high level of reactive oxygen species (ROS) by activating the cellular oxidative stress response, or (2) Dsup reduces ROS levels through an unknown mechanism. To distinguish between these two possibilities, we examined the DAF-16/FOXO signaling pathway, which is activated by oxidative stressors such as arsenite and juglone (*18*, *19*). Upon activation, DAF-16 translocates to the nucleus, inducing genes that promote cellular stress response (*20*, *21*). If Dsup constitutively or excessively activates this pathway, we would expect an increased DAF-16::GFP (green fluorescent protein) nuclear signal. However, while wild-type N2 worms exposed to 1 mM arsenite exhibit DAF-16 nuclear activation within 15 min, this activation is barely detectable in Dsup-expressing worms even after 2 hours (Fig. 2D, 0.25-hour time point). Moreover, for a higher arsenite concentration (5 mM), DAF-16 activation is strongly delayed in Dsup-expressing worms (fig. S1B). These results indicate that Dsup does not enhance DAF-16 activation but instead delays it, ruling out a mechanism involving increased oxidative stress response activation through the DAF-16 pathway. We next examined the ROS level directly in worms to test whether Dsup can affect the cellular H_2_O_2_ level, by using the HyPer biosensor (*22*, *23*). HyPer is derived from circularly permutated yellow fluorescent protein and OxyR, an *Escherichia coli* protein that specifically reacts with H_2_O_2_. When oxidized by H_2_O_2_, two key Cys residues form a disulphide bond that induces a conformational change, which increases at 500 nm and decreases at 420 nm in the excitation spectral profile when 530-nm emission is monitored. By scanning the whole animal for 405- and 488-nm excitation at 530-nm emission, the intracellular redox activity can be deduced from the HyPer ratio measure (Fig. 2E). In untreated young adults [24 hours post-L4 (larvae stage 4)], wild-type N2 and Dsup-expressing worms exhibit similar HyPer ratio, suggesting that Dsup does not affect basal redox homeostasis in well-fed young animals (Fig. 2E, right, control condition). However, following arsenite exposure, N2 worms showed a significant increase in oxidized HyPer, whereas Dsup-expressing worms maintained HyPer ratios comparable to untreated controls (Fig. 2E, right). Together, these findings demonstrate that Dsup confers strong oxidative stress resistance by reducing ROS levels rather than by enhancing the cellular stress response. The exact mechanism underlying this ROS-reducing effect remains unknown.

### Dsup extends *C. elegans* life span

**Fig. 3.**
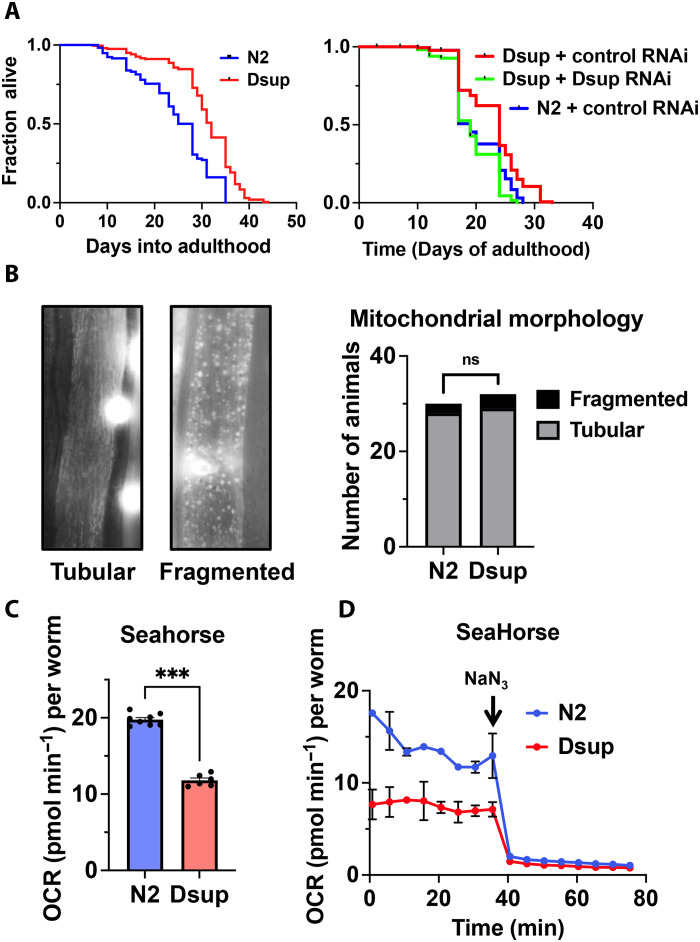
Dsup expression extends *C. elegans* life span by decreasing mitochondrial respiration. (**A**) Heterologous expression of Dsup extends *C. elegans* life span (left), and its silencing through RNAi suppresses the observed long-live phenotype (right), *n* = 60 in three independent experiments. (**B**) Mitochondrial morphology is not affected by the expression of Dsup. Representative image of mitochondrial tubular (normal) and fragmented morphology (left). Number of 24-hour post-L4 larvae stage animals with tubular or fragmented mitochondrial morphology (*n* = 32 and 31). (**C**) Dsup expression leads to a strong mitochondrial respiration decrease shown by the OCR, *n* = 200 in triplicate. Data are means ± SEM, *P* values were determined using two-tailed Student’s *t* test. (**D**) Sodium azide (NaN_3_) treatment completely abrogates OCR difference between N2 control and Dsup. The graph represents the aggregation of two separate experiments.

Although a clear and unified theory of aging is still lacking (*24*, *25*), oxidative stress has been proposed to contribute to the progressive loss of tissue and organ function during aging. Because Dsup expression in *C. elegans* provides a strong protection against oxidative stress–inducing agents, we examined whether physiological aging is affected by the presence of Dsup. *C. elegans* is a powerful and well-established model organism for the investigation of aging-related mechanisms, and numerous evolutionary conserved signaling pathways have been found by studying this process in the worm. The worm life span is increased by the expression of the Dsup gene (Fig. 3A), suggesting that the protection against oxidative stress provided by Dsup is related to a resistance to the aging process development. To confirm our findings, we performed a gene expression knock-down by RNAi and observed the significant suppression of the extended life span phenotype specifically in the situation when double-stranded RNA were designed to target Dsup (Fig. 3A, right graph). This result establishes that Dsup, when expressed in a multicellular organism, can extend life span presumably by protecting cells against endogenous oxidative stress that accumulates in aging animals.

### Dsup localization confirms nuclear accumulation in somatic cells

Next, we sought to determine Dsup localization by generating an mScarlet-Dsup fusion by CRISPR-Cas9. We confirmed that the mScarlet fusion does not interfere with Dsup function by validating the life span extension phenotype, similarly to our previous strain Dsup (fig. S2B). In larvae stage, Dsup is detected and localized at the nucleus of somatic cells, consistent with previous studies demonstrating a direct interaction between Dsup and the nucleosome (fig. S3A) (*15*, *26*, *27*). In late larvae stage (L4) Dsup is present in the intestine, body wall muscle, neuron, and vulva cell nuclei (fig. S3B). To our surprise, we could not detect Dsup signal in adult germ cells nor in early development embryonic cells (fig. S3C). In conclusion, although Dsup is expressed under a germline-specific promoter, it displays a strong somatic expression profile starting from early larvae stages to adult worms.

### Dsup delays DAF-16 activation during aging

Next, we sought to establish whether Dsup expression affects the primary signaling pathway involved in aging. The first *C. elegans* mutants identified with extended life spans, *age-1* and *daf-2*, encode phosphatidylinositol-3-OH kinase and an insulin-like receptor, respectively (*28*–*30*). Both function within the insulin/insulin-like growth factor-1 signaling (IIS) pathway. Either loss-of-function mutations in *age-1* or reduction-of-function mutations in *daf-2* leads to the activation of the DAF-16/FOXO transcription factor by inducing its translocation from the cytoplasm to the nucleus, where it activates prolongevity gene expression (*31*, *32*). Constitutive reduction-of-function of the IIS pathway leads to life span extension as well as resistance to various conditions such as oxidative stress, heat shock, heavy metals, hypoxia, and bacterial pathogens (*30*, *33*–*38*). To determine whether Dsup expression influences the IIS activation, we examined the transactivator DAF-16. In unstressed, well-fed young adult worms, DAF-16::GFP localization remains unchanged by Dsup expression (0-hour time point), indicating that Dsup does not phenocopy *daf-2* or *age-1* mutations, which constituvely activate DAF-16. During normal aging, DAF-16 translocates to the nucleus and activates age-associated genes in response to cellular stress (*39*). Since Dsup promotes life span extension without activating DAF-16, we analyzed its impact on DAF-16 status during aging. Consistent with previous findings (*39*), we observed DAF-16 activation in approximately half of wild-type worms five days after egg laying, with nearly complete activation by day 11 (fig. S2A). However, in Dsup-expressing worms, DAF-16 activation was substantially delayed, with only half of the population showing nuclear localization by day 11. This suggests that Dsup expression delays DAF-16 activation, not only in response to oxidative stress but also during the aging process.

### Dsup alters mitochondrial respiration and oxygen consumption

Apart from the IIS pathway, mutations in genes involved in mitochondrial functions can also lead to an extended life span in *C. elegans* (*40*–*42*). Impairment of the mitochondria functionality is a recognized characteristic of aging (*25*). The mitochondrial network structure greatly evolves during aging, switching from a highly organized and tubular structure in young animals, to a fragmented and globular pattern in old animals (*43*). In worms, several mutations cause mitochondria fragmentations and extended life span. These mutations affect genes involved in mitochondrial dynamics, stress response, and metabolic regulation (*30*, *44*). Since Dsup expression extends worms’ life span without inducing stress-responsive genetic program through DAF-16, we sought to test whether mitochondria were affected in Dsup-expressing worms. We first examined whether Dsup affects the overall mitochondrial network structure during the worm life span and could not observe any difference between N2 wild-type animals and the Dsup-expressing line (Fig. 3B). In *C. elegans*, partial loss-on-function mutations in the mitochondrial respiration chain complex subunits that reduce electron transport increase life span substantially. Thus, we sought to directly measure the mitochondrial respiration activity to ask whether Dsup interferes with the mitochondrial physiology. We adapted to *C. elegans* the Seahorse Extracellular Flux Analyzers that allow measurement of O_2_ consumption in multiple cells without the need to isolate mitochondria (*45*–*47*). The presence of Dsup induces an almost twofold decrease in the oxygen consumption rate (OCR) of young adult (Fig. 3C). To exclude that the lower OCR observed in Dsup is consequent to a nonmitochondrial activity, we performed a time-resolved analysis where sodium azide is supplemented to the medium. By blocking the mitochondrial respiration, sodium azide reveals nonmitochondrial oxygen-consuming processes such as oxygenases. The addition of sodium azide effectively blocks the OCR and notably abolishes entirely the difference between N2 control and Dsup worms (Fig. 3D), demonstrating that the lower oxygen consumption in the Dsup background is a consequence of a reduced mitochondrial respiration exclusively. Together, these results suggest that Dsup expression extend *C. elegans* life span by impeding mitochondrial respiration and promoting oxidative stress resistance.

## DISCUSSION

The ability of Dsup to extend *C. elegans* life span is intriguing for several reasons. Previously documented life span–extending interventions primarily involve genetic mutations in endogenous genes, exposure to specific compounds, or environmental modifications that affect metabolism regulation (*28*–*30*, *48*, *49*). The fact that Dsup, a tardigrade-specific gene, enhances animal fitness suggests that it either has an unidentified enzymatic function or modulated a conserved cellular pathway, requiring interactions with endogenous protein partners. Dsup-mediated life span extension occurs independently of the canonical DAF-2/DAF-16 signaling pathway and does not induce a constitutive stress-response state, unlike long-lived *daf-2* mutants. Furthermore, unlike germline ablation or mutations in germline proliferation genes such as *glp-1* (a *NOTCH* homolog), Dsup expression does not impair germline development or proliferation, ruling out reproductive signaling as the underlying mechanism of life span extension.

The observation that Dsup reduces mitochondrial respiration is particularly intriguing, given its known ability to bind and localize to nucleosomes (*15*). Unless a small, undetected fraction of Dsup is present in mitochondria—a possibility yet to be determined—this finding suggests that Dsup interferes with the mitochondrial electron transport chain through an unknown mechanism. While other mutations affecting mitochondrial respiration have been shown to extend *C. elegans* life span (*40*) and confer oxidative stress resistance [e.g., *isp-1* mutants (*50*)], these genetic changes typically come at the cost of reduced brood size (*51*), which is not observed in Dsup-expressing animals.

Our findings also demonstrate that Dsup can be heterologously expressed in a multicellular organism without causing widespread toxicity. This contrasts with previous studies reporting DNA damage in neurons upon Dsup overexpression (*52*). This discrepancy may stem from differences in expression levels: In our study, Dsup was stably integrated as a single copy under the control of a germline-specific promoter (spn-4p), whereas previous research used a strong cytomegalovirus promoter in neurons. However, we cannot rule out the possibility that Dsup may be inherently toxic to neurons, regardless of expression level. Notably, our initial attempts to drive ubiquitous Dsup expression using *eft-3p* were unsuccessful, suggesting that high expression levels may be detrimental in *C. elegans*. A similar phenomenon has been observed in *Drosophila*, where Dsup expression under the strong *Act-5* promoter confers stress resistance but impairs locomotion, indicating potential toxicity at high expression levels (*26*). In contrast, Dsup expression in plants like tobacco and rice enhances DNA damage resistance but does not extend life span or confer oxidative stress resistance (*14*, *53*), highlighting its context-dependent effects and sensitivity to expression levels.

In conclusion, our study demonstrates life span extension through heterologous expression of Dsup that does not occur at the expanse of a constitutive stress response, a mitochondrial genetic impairment, or a germline proliferation defect. These findings highlight a previously unknown mechanism of life span regulation and suggest that Dsup may interact with conserved cellular pathways beyond its established role in DNA protection.

## MATERIALS AND METHODS

### *C. elegans* culture methods and strains

General methods for worm handling were done as previously described (*54*, *55*). Wild-type *C*. *elegans* N2 and *E. coli* HT115 (DE3) strains were provided by the *Caenorhabditis* Genetics Center - CGC (funded by the National Institutes of Health National Center for Research Resources). All strains were maintained at 20°C and grown on nematod growth medium (NGM) plates seeded with *E*. *coli* HT115 (DE3). The following strains have been used: TJ356 [*daf-16p::daf-16a/b::GFP* + *rol-6* (*su1006*)], PD4251 [(*pSAK2*) *myo-3p ::GFP ::LacZ ::NLS +* (*pSAK4*) *myo-3p ::mitochondrial GFP +* dpy-20 (+)], JV1 [*rpl-17p ::HyPer + unc-119* (+)]

### Transgene constructs for the *C. elegans* strain expressing Dsup

The Dsup transgene was constructed following the MosSCI methods (service of In Vivo Biosystems Inc., UT. USA) (*16*). A 1329–base pair (bp) fragment of the full-length sequence encoding the *R. varierornatus* Dsup gene (GenBank accession LC050827) was cloned into the pNU3020 plasmid [*spn-4*::RvDsup::*unc-54u* in ttTi5605, *unc-119* (+)] used for injection. Isolated lines were confirmed by genotyping and sequencing.

Genomic integration of the Linker::wrmScarlet::3xFLAG fusion was outsourced by SEGiCel (SFR Santé Lyon Est, CNRS UAR 3453, Lyon, France), with the following oligo:

oMG270: AGAAGGGAGGAAAGGCtGGAGGTCGCAAGCGCAAAggaggaggtggaagcATGGTCAGCAAGGGAGAGGC

oMG271: AGAAGGGAGGAAAGGCtGGAGGTCGCAAGCGCAAAggaggaggtggaagcATGGTCAGCAAGGGAGAGGC.

### Dsup expression validation by RT-PCR

Gravid adults worms were collected from the plates and washed three times with phosphate-buffered saline to remove bacteria before introduction in TRIzol and frozen at −80°C. Total RNA was extracted by TRIzol/chloroform followed by RNeasy Kit (QIAGEN) and cDNA synthesis performed on 300 ng of RNA with the QuantiTech Reverse Transcription Kit (QIAGEN). RT-PCR reactions were performed with specific primers using the Phusion High-Fidelity DNA Polymerase (Thermo Fisher Scientific), and products were resolved on a 3% agarose gel. Tubulin (*tbg-1*) was used for internal control. The following primers were used:

Rv Dsup forward: GAGACAAGAAGGACGACTCC

Rv Dsup reverse: CCAAGGGAGGAGAGCAATC

*tbg-1* forward: AAGATCTATTGTTCTACCAGGC

*tbg-1* reverse: GTCAAGGACAAGAAGTTCAAG.

### Brood counts

Synchronized worms were cultured at 20°C. Brood counts were done by picking 10 young adult worms of either N2 wild-type control strain or Dsup-expressing strain to separate NGM plates. The number of eggs laid by each worm was counted every day until the end of the egg-laying period.

### Pumping

As previously described (*56*), pumping was measured by counting grinder movements using a stereomicroscope (Leica, M205FCA). Pumping rate was counted for 20 s every minute for 10 min per young adult nematode. Measurements were carried out on 10 worms per condition.

### Ionizing radiation sensitivity assay

About 30 24-hour post–L4 stage worms were individually picked in a fresh plate, exposed to the indicated ionizing radiation dose, and then incubated for 24 hours. Eight plates for each condition were incubated with five worms that were allowed to lay eggs for 12 hours before being removed. The total number of laid eggs per plate was determined before incubating the plates for another 24 hours. Last, the total number of larvae was determined, and the survival rate was calculated by dividing the number of larvae by the number of total eggs for each plate.

### Life span assay

Three independent life span experiments were performed at 20°C for all strains. For each experimental condition, up to 60 synchronized L4 larvae were transferred on a 96-well plate (five worms per well) together with *E. coli* HT115 (DE3) strain as food source in a final volume of 200 μl of S-Medium containing 5-Fluoro-2′-deoxyuridine (FUdr) to avoid offspring (*57*). Live worms were scored daily by counting using a soft touch with a platinum wire.

### Life span statistical analyses

Statistical analyses of life span were performed using XLStat statistical software (Addinsoft, New York, NY, USA). Calculation of the *P* values for the Kaplan-Meier log-rank test was performed with a 95% confidence level. Student’s *t* tests were performed with a 95% confidence level.

### Oxidative stress assay using H_2_O_2_

For either the Dsup or the N2 control strain, 100 synchronized L4 larvae were transferred on a 24-well plate and then treated with or without 1 mM H_2_O_2_ for 30 min at 20°C. The survival rate was scored 16 hours later. This experiment was performed in triplicate.

### Oxidative stress assay with methyl viologen (paraquat)

For the analysis of both Dsup and the N2 control strain, 50 synchronized L4 larvae were added to 24-well plates and treated with or without 200 mM paraquat (methyl viologen dichloride hydrate, 856177, Sigma-Aldrich, Saint Louis, MO, USA) for 24 hours at 20°C. Survival rate was assessed every hour during a time lapse of 6 hours and then after 24 hours. This experiment was performed in triplicate.

### Hydroxyl radical oxidative stress by using the Fenton reaction

Hydroxyl radical treatment was performed by using a modification of the Fenton reaction (*58*). Groups of 50 young adult *C. elegans* were dropped in a 1.5-ml Eppendorf tube containing 24 μl of S-Medium at room T° (20°C). A 3.3-μl droplet of EDTA 200 μM and 100 μM Fe (II) solution was added beneath the cover, and then a 3.3-μl droplet of 30% H_2_O_2_ was added at the opposite side. Hydroxyl radical production was initiated by a brief spin (6,000 RPM for 5 s). The incubation (30 s) was stopped by adding 1 ml of S-Medium followed by a short spin (6,000 RPM for 5 s) and a second rinse with 1 ml of S-Medium. Worms were transferred onto a clear NGM agar plate and counted for survival 3 hours later. This experiment was performed in triplicate.

### DAF-16 activation upon arsenite treatment

At least 20 young adult worms (24-hour post-L4 stage) were picked on a 6-cm 1× NGM plate supplemented with arsenite (1 mM final concentration) and assessed for DAF-16 activation by scoring the number of animals with DAF-16::GFP nuclear localization using a fluorescent stereoscope (Leica, M205FCA) at the indicated time.

### DAF-16 activation during *C. elegans* life span

Between 100 and 150 synchronized L4 larvae were distributed in 1× NGM plates and transferred on fresh plates every two days to separate offspring’s. At least 20 worms were mounted for microscopic observation every 2 days on a 3% agarose pad melted in M9 buffer. DAF-16 activation was assessed by direct observation with an epifluorescent upright widefield microscope (Leica upright, 40×immersion oil objective).

We noticed that when worms are mounted for microscopic observation, DAF-16 autoactivation occurs within 5 min; therefore, worms that are not assessed for DAF-16 localization within 3 to 4 min were censored.

### RNAi

The plasmids used for RNAi experiments are derivative of the L4440 backbone (*59*). For Dsup RNAi, 300 bp of the N-terminal Rv Dsup gene was cloned into the L4440 vector with the following primers:

5′ to 3′ forward: TATATAGCGGCCGCATGGCCTCCACCCACCAATCCTCCACC

5′ to 3′ reverse: ATATATGCGGCCGCGGACTTCTCCTTTTGGTCGG

A plasmid containing the GFP sequence with a worm-optimized intron was used as a control.

### RNAi knockdown

Synchronized L4 larvae fed with RNAi-producing bacteria were allowed to grow at 20°C on supplemented NGM plates, including FUdR to avoid offspring (*57*). Worms were counted daily for touch-provoked movement. Dead worms were scored, and worms that crawled off the plate or displayed extruded internal organs (bagging phenotype) were censored. The life span assays were performed tree times.

### In vivo OCR measurements

OCR measurements were performed on a Seahorse XFe96 Extracellular Flux Analyzer (Seahorse bioscience Inc., North Billerica, MA, USA) as previously described (*47*, *60*) but with the following modifications. Synchronized young adult worms were washed three times and starved for 12 hours in S-Medium to allow complete elimination of gut bacteria. Groups of 25 worms were disposed in each well of the 96-well plate of the Seahorse XF96 analyzer in a final volume of 200 μl S-Medium. Internal control measurements were done by filling eight wells with 200 μl of S-Medium. The computer program used was as follows: 1, calibrate probe; 2, loop 64 times; 2a, mix for 2 min; 2b, time delay 30 s; 2c, measure 3 min; 3, loop end. The internal heater of the device was set off. This experiment was conducted three times. In a second time, to completely block mitochondrial respiration, sodium azide was injected at the end of the assay in a final concentration of 40 mM per well. The computer program used was as follows: 1, calibrate probe; 2, loop eight times; 2a, mix for 2 min; 2b, time delay 30 s; 2c, measure 2 min; 3, loop end; 4, inject port A/sodium azide; 5, loop eight times; 5a, mix for 2 min; 5b, time delay 30 s; 5c, measure 2 min; 6, loop end; 7, program end. The internal heater of the device was set off. This experiment was conducted three times.

### Motility

Motility was measured following the radial locomotion assay (*61*, *62*). Briefly, young adult worms were picked in the center of a plate, and radial distance traveled was measured 10 min later.

### HyPer biosensor

The H_2_O_2_ biosensor HyPer was used according to previous study. Briefly, young adult worms exposed to the specified treatment were immobilized on a 3% agarose pad in M9 buffer supplemented with 5 mM levamisole and imaged with Confocal Zeiss LSM880 Airyscan. Images of the worms were acquired using Confocal Zeiss LSM980 NLO using 10× EC Plan Neofluar 0.3–numerical aperture objective. The images of worms were recorded using Zeiss Zen Blue software 3.5 in tile format. Images were stitched using Zeiss Zen Blue software 3.5 before being analyzed. Images were segmented by thresholding method using Fiji ImageJ ver 1.53t. We used the moments algorithm as a global thresholding method along with a dark background as an additional option. Segmented images covering the worm body were analysed to measure the integrated density using Fiji ImageJ.

### *mScarlet-Dsup* in vivo detection

Worms from a growing population were mounted for microscopic observation on a 3% agarose pad melted in M9 buffer. mScarlet was detected with an epifluorescent upright widefield microscope (Zeiss AxioImager Z2) using ZEN software (version 3.4.91, Blue edition). Representative images are presented following max-project of Z-stacked images using maximum intensity projection type with Fiji ImageJ software (version 2.3.0/1.53f).
