# Supplementary material for: Tardigrade Dsup extends C. elegans life span by impeding mitochondrial respiration and promoting oxidative stress resistance
Source: Sci Adv. 2025 Oct 31;11(44):eadx9669. doi: 10.1126/sciadv.adx9669 (PMC12577708; doi:10.1126/sciadv.adx9669)
Supplement: Supplementary file 1 — Figs. S1 to S3 [file sciadv.adx9669_sm.pdf]

Science Advances
AAAS

Supplementary Materials for

Tardigrade Dsup extends *C. elegans* life span by impeding mitochondrial respiration and promoting oxidative stress resistance

Myriam Richaud *et al.*

Corresponding author: Simon Galas, simon.galas@umontpellier.fr; Aymeric Bailly, aymeric.bailly@crbm.cnrs.fr

Sci. Adv. **11**, eadx9669 (2025)
DOI: 10.1126/sciadv.adx9669

This PDF file includes:

Figs. S1 to S3

Supplementary Figures

Fig. S1.

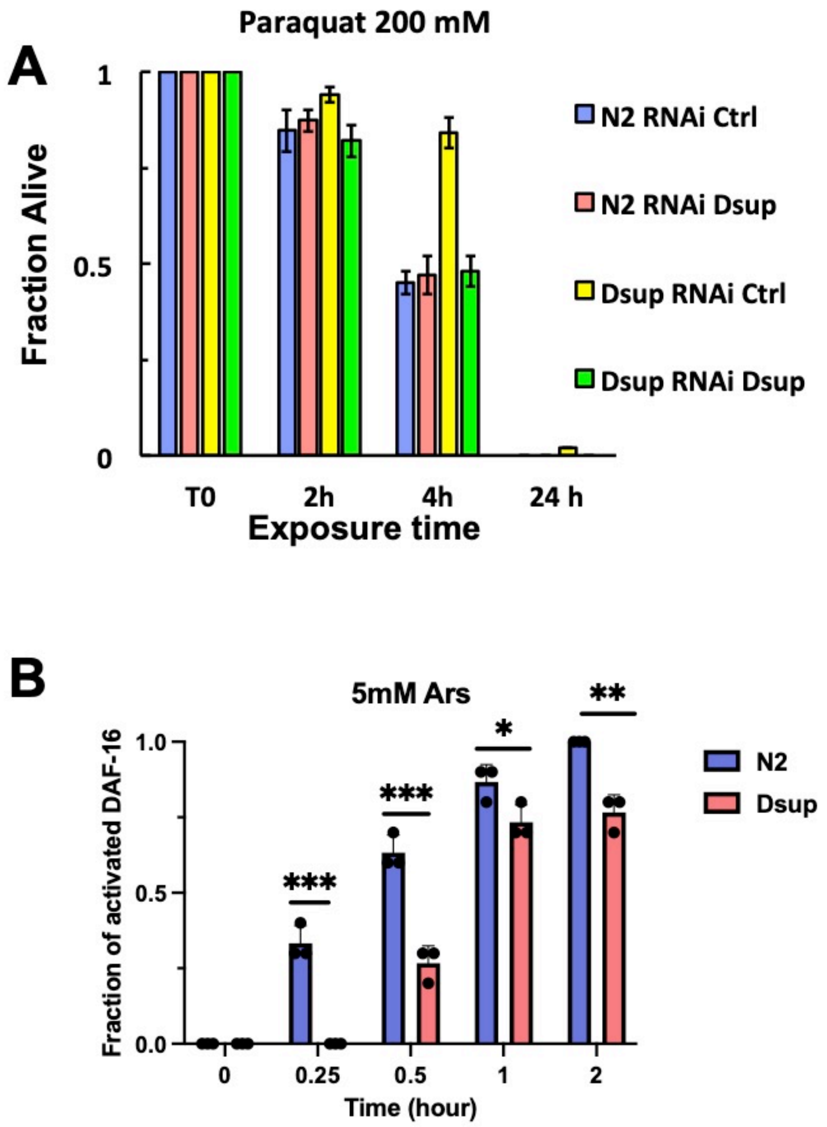

Figure S1: Dsup confers resistance to Paraquat and delays DAF-16 activation upon Arsenite treatment.

A. Fraction of surviving animals of the indicated genotype following treatment of L4 larvae stage worms (*n* = 50 in triplicate) with 200 mM of paraquat (methyl viologen). Data are mean ± s.e.m.

B. Dsup expression delays DAF-16 activation induced by Arsenite exposure. DAF-16 activation ratio was assessed by quantifying the fraction of animals with DAF-16::GFP nuclear versus cytoplasmic localization exposed to 5 mM of Arsenite, similarly to Fig. 2B. Data are mean ± s.e.m. *P* values were determined using two-tailed Student's *t*-test (*n* = 30, in triplicate).

Fig. S2.

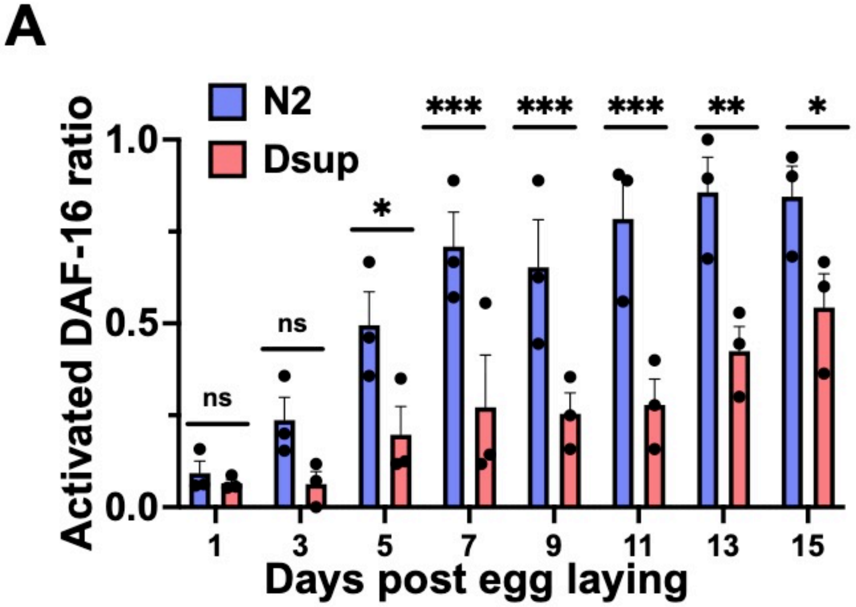

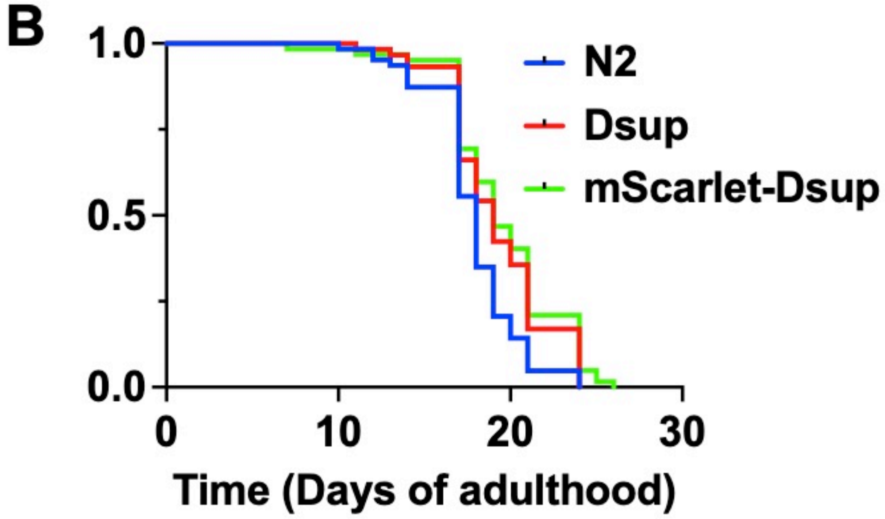

Figure S2: Dsup expression protects from oxidative stress.
A. Dsup expression delays DAF-16 activation induced by Arsenite exposure. DAF-16 activation ratio was assessed by measuring the fraction of animals with DAF-16::GFP nuclear versus cytoplasmic localization exposed to 5 mM of Arsenite. Data are mean ± s.e.m.. *P* values were determined using two-tailed Student's *t*-test, *n* = 30, in triplicate.
B. mScarlet fusion does not interfere with Dsup lifespan extension phenotype. Lifepsan analysis was performed on *n* = 60 worms in three independent experiments.

Fig. S3.

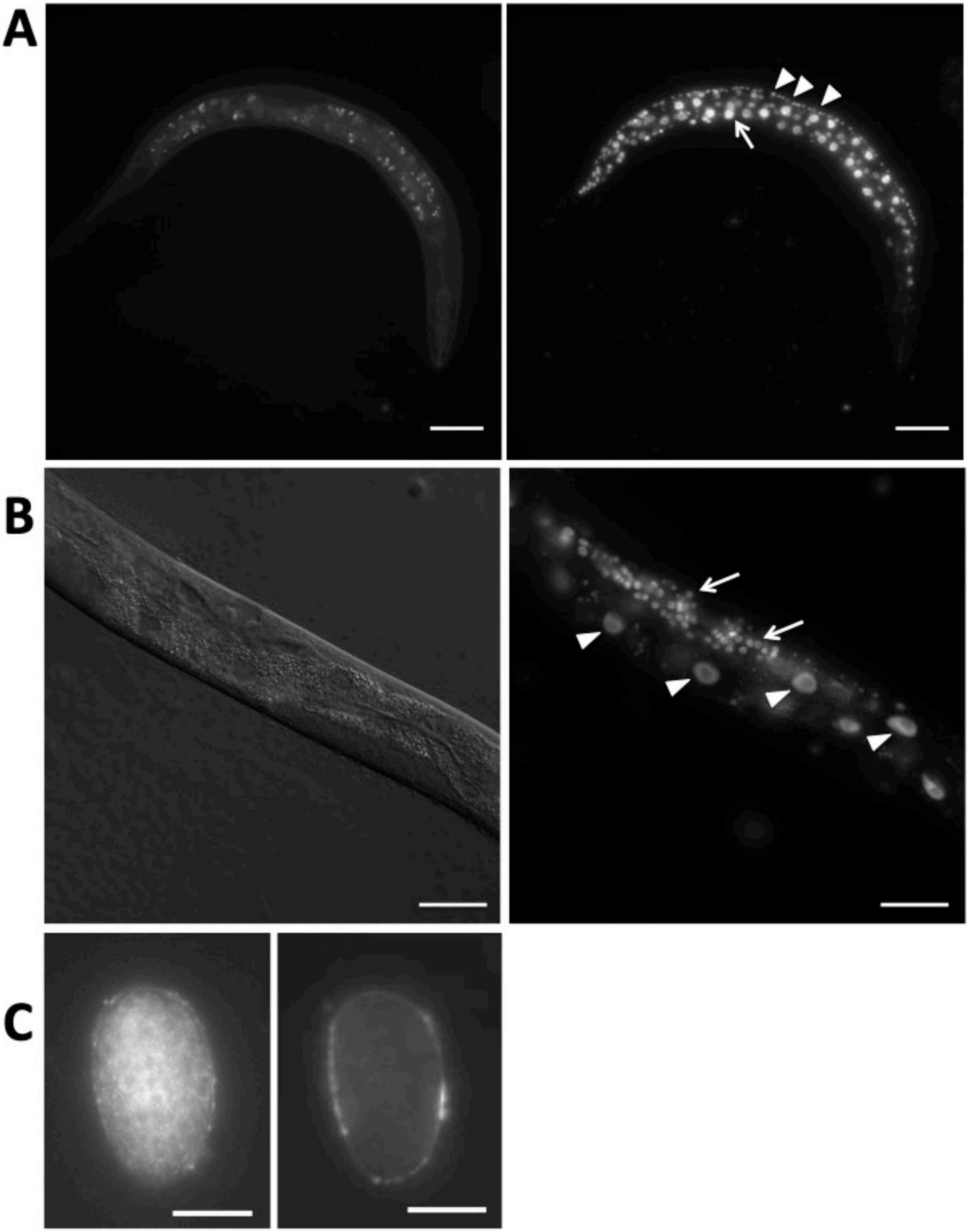

Figure S3: Dsup is expressed in somatic cells during larvae development.
A. Dsup expression is detected in somatic cell nuclei during larvae stages. Representative L1-stage worm showing mScarlet-specific signal. Left panel: autofluorescence signal (green channel). Right panel: mScarlet signal (red channel). White arrowheads indicate neurons (dorsal nerve cord), while white arrows indicate intestinal cell nuclei. Scale bar = 40 μm.
B. In L4-stage larvae, Dsup is present in intestinal cells, body wall muscle (white arrowheads), neurons and vulva cell nuclei (white arrows). **C.** mScarlet-Dsup is not expressed during early embryo development stages. Representative embryo image shown. Left panel: autofluorescence signal (green channel). Right panel: mScarlet-specific signal (red channel). Scale bar = 25 μm.
